# Glycosylation and Acylation: Important Regulators of Immune Cell Fate Decisions

**DOI:** 10.3390/biology14060611

**Published:** 2025-05-27

**Authors:** Han Wang, Yiying Zhang, Xu Luo, Xinxin Zheng, Guangdong Bai, Junhui Liu

**Affiliations:** College of Animal Science and Technology, Southwest University, Chongqing 400715, China; wh04032024@outlook.com (H.W.); zyy040822mkkk@outlook.com (Y.Z.); l.xu1113@outlook.com (X.L.); xinxin262424@outlook.com (X.Z.)

**Keywords:** immune cell fate, glycosylation, acetylation, succinylation, crotonylation, immune diseases

## Abstract

Dissecting the determinants of immune cell fate is a central challenge in immunology and is important for understanding cell differentiation, disease diagnosis, and therapy. Post-translational modifications (PTMs) of proteins are chemical modifications of amino acids involving the addition or removal of specific groups. As molecular gatekeepers of immune cell fate, PTMs affect immune function mainly through the regulation of important life processes such as immune cell growth, proliferation, differentiation, activation, and apoptosis. Among PTMs, glycosylation and acylation have emerged as critical regulatory mechanisms governing immune homeostasis. Through precise structural alterations of immune-related proteins and receptors, glycosylation shapes antigen recognition, cytokine signaling, and intercellular communication, while acylation regulates signal transduction and epigenetic modifications in both innate and adaptive immunity. These modifications are involved in the regulation of a wide range of biological processes and are intricately associated with the pathogenesis of many diseases, especially immune diseases. Therefore, this paper reviews the functions and mechanisms of glycosylation and acylation in regulating innate and acquired immunity, to provide new insights into the role of PTMs in disease pathogenesis and potential targeted therapies.

## Simple Summary

This paper systematically reviews the mechanisms of glycosylation and acylation modifications in immune regulation. It breaks through the limitations of traditional single-modification studies and innovatively establishes the functional association between the metabolic microenvironment and PTMs. Potential clinical applications are prospectively envisioned.

## 1. Introduction

**Figure 1:**
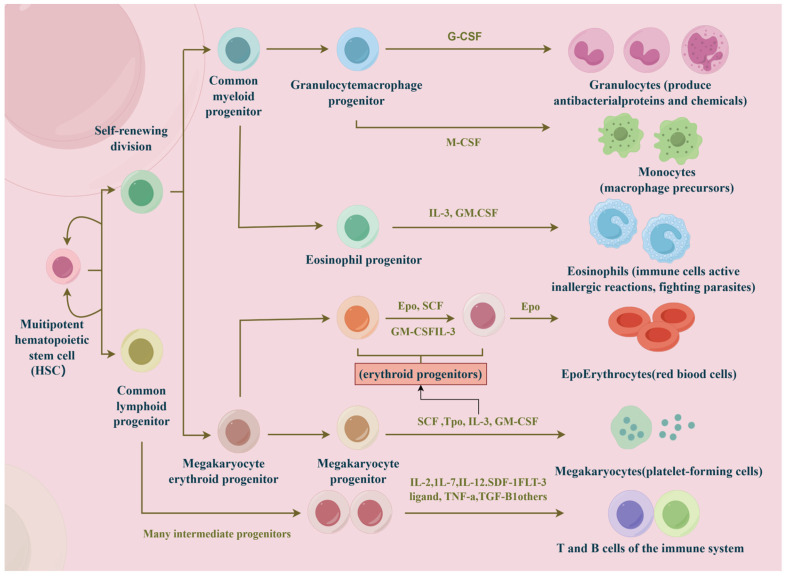
The main types of immune cells. Hematopoietic stem cells differentiate into two subgroups, that is, myeloid and lymphoid cells. Under the influence of cytokines, they further differentiate into macrophages, mast cells, neutrophils, T cells, B cells, natural killer (NK) cells, and dendritic cells.

Deciphering the determinants of immune cell fate has been a persistent challenge in the field of immunology. Unraveling these complex mechanisms not only clarifies the fundamental principles of cell differentiation and development but also provides critical insights into the origins of diseases such as cancer. Immune cells, commonly known as white blood cells, are essential to the immune response. They can be broadly categorized into two main subgroups, that is, myeloid cells and lymphoid cells, both of which are derived from multipotent hematopoietic stem cells. Key types of immune cells include macrophages, mast cells, neutrophils, T cells, B cells, natural killer (NK) cells, and dendritic cells (Figure 1) [1].

Protein post-translational modifications (PTMs) usually refer to the enzymatic or chemical alteration of chemical groups on the amino acid residues of proteins during or after their biosynthesis, thereby changing the properties and functions of proteins. PTMs of proteins are diverse, and more than 500 types of modifications have been identified, with phosphorylation, ubiquitination, methylation, acetylation, glycosylation, etc., being the most common ones, generally resulting in a fixed molecular mass shift of the amino acid residues of proteins. The biological functions of PTMs include protein degradation and quality control, regulation of gene expression, enzyme activation, immune regulation, etc. [2]. In particular, regulating the fate of immune cells through the biological functions of PTMs to maintain the immune homeostasis of the organism is an important direction for future disease research. PTMs govern immune cell fate through hierarchical mechanisms, as follows:

(1) Gatekeeping development: For example, glycosylation of the pre-T-cell receptor (pre-TCR) filters autoreactive thymocytes via calnexin-mediated quality control [3].

(2) Dynamic reprogramming: Inflammatory stimuli (e.g., LPS) induce signal transducer and activator of transcription 6 (STAT6) succinylation, locking macrophages in immunosuppressive M2 states [4].

Thus, PTMs act as molecular switches at critical fate-decision points in immune cells within the body.

Currently, research on the role of PTMs in regulating immune checkpoints is expanding [5]. For example, in breast cancer cells, PD-1/PDL1 interaction enhanced Akt and ERK phosphorylation, leading to activation of the PI3K/AKT and MAPK/ERK pathways and increased MDR1/P-gp expression. This interaction simultaneously increased the survival of adriamycin-treated breast cancer cells, suggesting that inhibition of PD-1/PD-L1 may enhance the efficacy of chemotherapy in a non-immunological manner [6]. In a CRC model, a mannosidase inhibitor blocked galectin-9 binding to TIM-3 and restored the cytotoxicity of CD8+ T cells [7,8], suggesting promising applications in immunotherapy.

In this review, we provide a comprehensive overview of how the regulation of glycosylation and acylation influences immune cell fate, highlighting the mechanisms by which these modifications influence immune cell differentiation, function, and response to external stimuli. This research could provide insights into the treatment of immune diseases and the development of therapeutic protein drugs.

## 2. Regulation of Glycosylation in Immune Cells

Glycosylation is the process by which enzymes attach sugars to proteins [9]. Glycosylation is observed in various forms, with N-glycosylation and O-glycosylation being the most extensively studied types. N-glycosylation occurs when sugar chains are attached to the amino acid asparagine, typically at a specific sequence, e.g., Asn-X-Ser/Thr. These sugar chains critically modulate protein function and interactions. O-glycosylation is highly variable, including N-acetylglucosamine, fucose, and mannose. Due to its complexity, this form of glycosylation is difficult to predict [10]. We will discuss how glycosylation regulates immune cells and their mechanisms (see Table 1).

### 2.1. Innate Immune Cells

Changes in glycosylation influence the migration, activation, and interactions of innate immune cells, which subsequently impact the inflammatory response. In the circulation, neutrophils normally remain quiescent to prevent premature activation in the bloodstream, leading to systemic organ failure or even death [30]. Sialic acid-binding immunoglobulin-like lectin 9 (siglec-9) on erythrocytes interacts with glycoproteins on the surface of neutrophils and helps stabilize the activated state of neutrophils through downstream inhibition of signaling pathways [31].

#### 2.1.1. Neutrophils

As neutrophils migrate to inflammation sites, they migrate from the circulation to surrounding tissues through a cascade of leukocyte adhesion reactions dependent on glycosylation and the glycan-binding protein (GBP) [32]. The endothelial cells lining the blood vessels near inflamed sites express two key cell adhesion molecules (CAMs), P-selectin (P16109) and E-selectin (P16581), upon inflammation-associated cytokine stimulation. Both P-selectin and E-selectin bind P-selectin glycoprotein ligand-1 (PSGL-1, Q14242), a glycoprotein expressed on the surface of neutrophils, which facilitates neutrophil ‘tethering’ [11]. Neutrophil functions at sites of inflammation, including phagocytosis, degranulation, and the formation of neutrophil extracellular traps (NETs), rely on interactions with innate and adaptive immune cells [33]. Glycosylation modifications of neutrophils may facilitate communication between these cells [34]. For example, azurocidin, secreted via N-glycan mannose-6-phosphate modification, binds to the macrophage surface mannose receptor (MRC1) and promotes monocyte chemotaxis [12,13]. The secondary granule protein LCN2, through terminal α2–6 sialylation modification, can bind to dendritic cell Siglec-5 to inhibit excessive inflammatory response [12,35]. Additionally, glycoRNAs [14] on the surface of neutrophils are essential for regulating the inflammatory response of neutrophils. Neutrophil glycoRNAs are mainly located on the extracellular surface and can be recognized by P-selectin expressed by endothelial cells. Lu et al. experimentally found that the elimination of cell-surface RNAs significantly reduced neutrophil recruitment to inflammatory sites in vivo in mice, as well as reduced neutrophil adhesion to and migration from endothelial cells, highlighting the critical role of glycoRNAs [15].

#### 2.1.2. Natural Killer (NK) Cells

Natural killer (NK) cells are known for their ability to kill infected or cancerous cells and secrete pro-inflammatory cytokines. Sialylation, a specific type of glycosylation, is indispensable for regulating NK cell activity [36]. This process involves the addition of sialic acid to glycoproteins and serves as a key modulator of NK cell activity by balancing activation and inhibition. For instance, Siglec-7 and Siglec-9 are inhibitory receptors on NK cells that bind to sialylated molecules. Siglec-7 primarily recognizes gangliosides on tumor cells [16], while Siglec-9 exhibits a high affinity for α2–6 and α2–3 sialylated molecules, including sialyl-Lewis X (sLex) [17]. Upon binding to sialylated glycans on target cells, these receptors transmit inhibitory signals to NK cells, reducing their ability to kill the target. Conversely, FcγRIIIa (CD16a), the most abundant activating receptor on NK cells, displays enhanced binding to pro-inflammatory IgG antibodies when it carries oligomannose-type N-glycans. However, this binding affinity is reduced when the receptor is heavily sialylated. It severely affects the function of NK cells. Cytokines such as interleukin-2 (IL-2) and interferon-α (IFN-α) have been shown to increase the sialylation levels on NK cell surfaces, potentially modulating receptor function and the overall activity of NK cells [18].

#### 2.1.3. Macrophage

The migration of monocytes through endothelial layers, their differentiation into macrophages, and activation at sites of inflammation are all critical steps in the immune response. These processes are heavily reliant on interactions with N-glycosylated adhesion molecules, such as ICAM-1, VCAM-1, and E-selectin. Notably, the expression and glycosylation states of these adhesion molecules can be modulated by pro-inflammatory cytokines like TNF-α [13,19]. The initial step in the immune response involves the recruitment of monocytes to sites of inflammation. This process is guided by chemokines such as MCP-1 and is critically dependent on complex interactions with highly N-glycosylated adhesion molecules. TNF-α, a key pro-inflammatory cytokine, has been shown to upregulate the expression of these adhesion molecules and alter their N-glycosylation status, thereby facilitating the trans-endothelial migration of monocytes [19,35]. Once monocytes have migrated across the endothelial layer, they differentiate into macrophages. This differentiation process is also influenced by changes in N-glycosylation. For example, D-mannose has been shown to inhibit LPS-induced macrophage polarization by suppressing IL-1β production, highlighting the importance of glycosylation modifications in shaping macrophage function. The activation of macrophages at the site of inflammation is another critical step in the immune response. This process is directly affected by N-glycosylation. For instance, lectins such as DC-SIGN (dendritic cell-specific intercellular adhesion molecule-3-grabbing non-integrin) on macrophage cells can bind to fucosylated sugars, triggering the release of anti-inflammatory molecules and dampening the immune response [10]. Additionally, glycoproteins like CD47 and CD24, which are highly expressed on tumor cells, interact with inhibitory receptors on macrophages (such as SIRPα and Siglec-10) to inhibit phagocytosis. Siglec-10, in particular, tends to bind to sialylated complex-type N-glycans on the substrate [20].

### 2.2. Acquired Immune Cells

#### 2.2.1. T Cells

In the thymus, T-cell development undergoes a series of stages. Firstly, thymic seeding progenitors (TSPs) migrate to the thymus through the interaction of P-selectin with its ligand PSGL-1. α1,3-fucosylation of PSGL-1 is critical for its binding to P-selectin, and impaired fucosylation disrupts TSP homing, compromising TSP colonization and subsequent differentiation into early T-cell precursors (ETPs) [37]. During the double-negative (DN) stages, ETP cells rearrange the TCRβ gene with the help of the RAG complex to form a pre-TCR complex. Glycosylation ensures the proper folding, stability, and surface expression of the pre-TCR complex. Successful expression of the pre-TCR is crucial for progression to the DN4 stage and subsequent differentiation into double-positive (DP) T cells [3,22]. DN3 thymocytes enter the β-selection phase after TCRβ rearrangement and must generate UDP-GlcNAc through the hexosamine biosynthetic pathway (HBP), which provides a substrate for O-GlcNAc transferase (OGT) [38]. OGT-mediated O-GlcNAcylation regulates c-Myc stability and promotes clonal expansion of DN4 cells; the absence of OGT leads to a reduction in DP cells [39].

**Figure 2:**
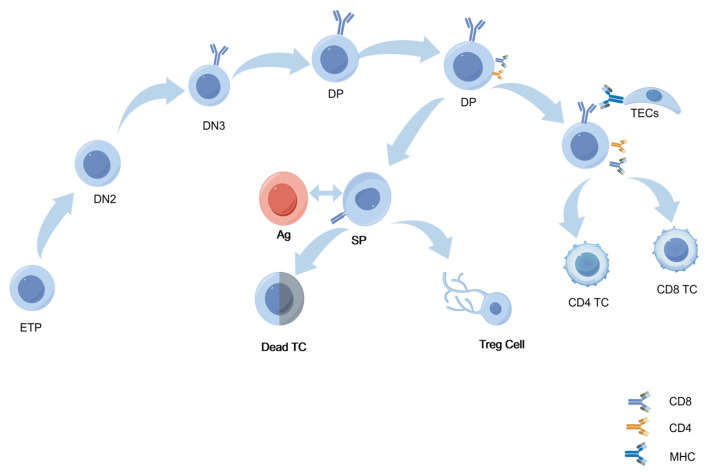
Glycosylation in the thymic microenvironment regulates key stages of T-cell development. Early thymic progenitor cells (ETPs) sequentially progress through the double-negative (DN) stages, especially DN2 and DN3, to develop into double-positive (DP) T cells. N-glycosylation of the pre-T-cell receptor (pre-TCR) facilitates proper receptor folding and initiates β-selection. β-selected cells upregulate CD4/CD8, entering DP stages to interact with thymic epithelial cell (TEC) MHC–self-peptides. Glycosylation (e.g., O-GlcNAc) modulates TCR signaling to set selection thresholds, enabling DP cells with appropriate MHC–peptide affinity to survive and differentiate into CD4+/CD8+ single-positive (SP) cells. High-affinity SP cells undergo apoptosis or become Tregs; non-binding SP cells mature and enter peripheral immunity.

Glycosylation continues to be significant in the DP stage, and T cells mature further by upregulating CD4 and CD8 and assembling a mature TCR [40]. Thymic epithelial cells (TECs) then assess these TCRs for their ability to recognize specific peptide–MHC complexes through positive and negative selection [41]. Glycosylation, especially N-glycosylation, fine-tunes TCR affinity for self–MHC peptides by modulating the spatial conformation of the TCR αβ chain. During positive selection, T cells that can recognize self–MHC peptides receive survival signals and differentiate into CD4 or CD8 single-positive (SP) T cells [40]. In Gfat1 knockout models, the deficiency of UDP-GlcNAc leads to reduced surface expression of TCRβ and CD5, impacting positive selection [42]. Moreover, experiments by Manuel et al. have shown that O-GlcNAcylation levels increase during positive selection. When OGT is knocked out at the DP stage, this process is impaired, resulting in a decrease in the number of mature SP cells [21]. Negative selection is another crucial step. Appropriate glycosylation maintains the structural integrity of the antigen-presenting complex, thereby ensuring efficient clearance by high-affinity self-antigen TCR T cells. However, this glycosylation-dependent process can be disrupted by the absence of *Mgat1* and potentially lead to autoimmune disorders [22,23] (Figure 2).

T-cell differentiation and activation are also influenced by glycosylation [35,43]. Studies have revealed that regulatory T cells (Tregs) exhibit abundant surface expression of N- and O-glycosylated IL-2 receptor α chains (CD25), suggesting that T cells require HBP-mediated glycosylation to facilitate membrane localization of IL-2 receptors, thereby enabling their differentiation into Treg cells [24].

PD-1, a protein on activated T cells, binds to its ligand PD-L1 to inhibit T-cell activation, a process of significant importance in tumor therapy research [25]. PD-1 is extensively N-glycosylated in T cells, with its extracellular domain containing the following four N-glycosylation sites: N49, N58, N74, and N116. Blocking the core fucosylation of N49 and N74 by inhibiting Fut8 reduces PD-1 expression and enhances T-cell activation [26]. D-mannose glycosylation degrades PD-L1 proteins and enhances T-cell function in the tumor environment. It impairs PD-L1 protein glycosylation by activating AMPK, which promotes PD-L1 degradation through the proteasome pathway and ultimately leads to PD-1 dysfunction. In T-cell-mediated tumor cell killing assays, pretreatment of activated CD8+ T cells with D-mannose significantly enhances their ability to kill tumor cells [43]. In addition to the PD-1/PD-L1 pathway, glycosylation also regulates other receptor–ligand interactions, such as the binding of co-stimulatory CD28 to CD80 and CD86. Specifically, N-glycosylation reduces the binding affinity of CD28 for CD80, thus acting as a brake on T-cell activation [26].

Antibodies targeting PD-1/PD-L1 have become a focus of clinical research given the critical role of PD-1 glycosylation on T-cell function. One has witnessed the simultaneous development of several different anti-PD-1 and anti-PD-L1 antibodies in multiple cancer types. Among these drugs, the FDA has approved two PD-1 antibodies (pembrolizumab and nivolumab) and three PD-L1 antibodies (atezolizumab, avelumab, and durvalumab) for use in cancer treatment [44].

#### 2.2.2. B Cells

Similar to T cells, glycosylation affects B cells at every stage from early maturation to differentiation into antibody-secreting plasma cells. During the process of early B-cell development, N-linked glycosylation of the μ-heavy chain is essential for proper pre-B-cell receptor (pre-BCR) assembly. The absence of this glycosylation impairs the development of B cells [27]. Studies have shown that galectin-1, a sugar-binding protein produced by bone marrow stromal cells, interacts with glycosylated integrins on pre-B cells, facilitating pre-BCR clustering and thereby promoting efficient B-cell maturation [28]. As these pre-B cells expand, the pre-BCR signaling triggers DNA rearrangements at the light chain loci, resulting in the formation of the mature B-cell receptor (BCR). If BCR binds self-antigens, immature B cells die as a result of negative selection, whereas cells showing tolerance to BCR are positively selected, resulting in germinal center B-cell tolerance [27].

**Figure 3:**
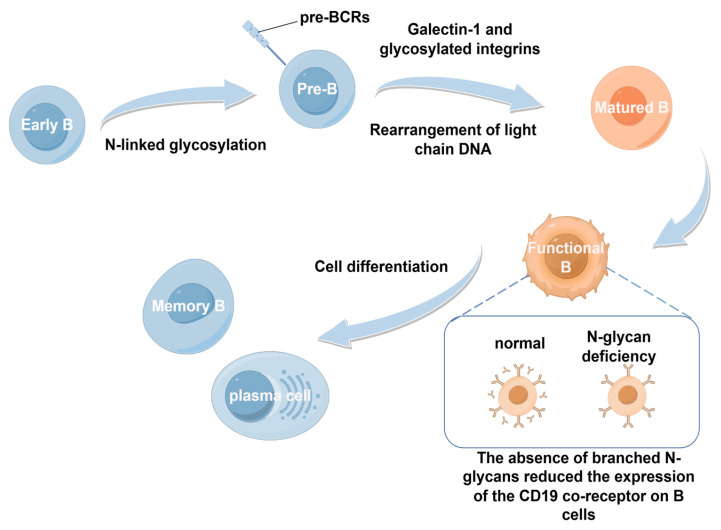
Developmental process of B cells. Early B cells undergo N-linked glycosylation to become pre-B cells. Under the action of galectin-1 and glycosylated integrins, they undergo light-chain DNA rearrangement to become mature B cells, which then differentiate into functional B cells. Functional B cells give rise to memory B cells and plasma cells. During this process, the absence of N-glycans reduces the expression of the CD19 co-receptor on B cells, affecting the functions of B cells.

In addition, immature B cells further develop into fully mature B cells when entering the spleen. During this period, glycosylation, particularly N-glycan branching, is crucial in regulating positive and negative selection processes. For instance, studies using B-cell-specific *Mgat1* knockout mice revealed that the absence of branched N-glycans reduced the expression of the CD19 co-receptor on B cells, thereby disrupting positive selection and preventing the development of functional B cells [29]. Notably, the impact of glycosylation extends beyond this point. In the spleen, activated B cells differentiate into either memory B cells or plasma cells based on the antigens they encounter. Throughout this process, glycosylation of surface receptors plays a continuous role in regulating their interactions and survival [45,46] (Figure 3).

### 2.3. Clinical Studies on Glycosylation

Glycosylation modifications are mainly reflected in their dual applications as diagnostic biomarkers and immunotherapeutic targets in clinical practice. On the one hand, through the specific recognition of the glycosylated subtype AFP-L3 (AFP-L3%) of alpha-fetoprotein (AFP) by Lens culinaris lectin, it has been proven in practice that its sensitivity to early hepatocellular carcinoma (HCC) with a diameter of ≤2 cm is approximately 35–39%, and the specificity can reach over 90% [47]. AFP-L3% and AFP can be used for the risk assessment and screening of HCC in high-risk patients with liver disease [47,48].

On the other hand, the vaccine Theratope^®^ (STn-KLH), based on the abnormal O-glycosylated epitope sialyl-Tn (STn) on the surface of tumor cells, through conjugation with keyhole limpet hemocyanin (KLH) and cyclophosphamide pretreatment, successfully induced high-titer anti-STn IgG in phase II/III clinical trials. Moreover, it showed a potential trend toward improved survival in patients with high immune responses, fully demonstrating the feasibility and promising prospects of glycosylated antigens as immunotherapeutic targets [49].

In a prospective clinical study of patients with chronic lymphocytic leukemia (CLL), investigators established a flow cytometry-based O-GlcNAcylation assay platform to analyze peripheral blood lymphocytes from healthy controls and CLL patients and found that in CLL patients, the level of O-GlcNAc was significantly and positively correlated with total leukocyte count and lymphocyte count, whereas in healthy subjects it was not. This study suggests that O-GlcNAcylation can be used as a dynamic monitoring indicator for malignant hematologic diseases such as CLL to assess disease burden and the rate of progression, thus laying the foundation for the development of clinical diagnostic kits or biomarkers based on O-GlcNAc modification [50].

In another study involving 79 untreated and 24 ibrutinib-treated CLL patients, sialylation levels of cell surface and CD49d proteins were assessed using flow cytometry and Western blot. CLL cells with a highly “activated” phenotype were found to have significantly increased sialic acid modifications, and removal of sialic acid inhibited sialylation. Removal of sialic acid inhibited their ability to migrate, and regulation of cell surface glycosylation may be a novel strategy to inhibit tumor invasion [51].

## 3. Regulation of Acylation in Immune Cells

Similar to glycosylation, acylation can occur with or without enzymes, with enzymatic acylation being the more common mechanism. In this process, acyltransferases add acyl groups from coenzyme A to amino acids like lysine, glycine, cysteine, or serine on proteins, while deacylases remove them [52]. The acylation can be divided into crotonylation, malonylation, succinylation, lauroylation, and both short- and long-chain fatty acid modifications, such as myristoylation and palmitoylation [53]. In this section, acylation-based modifications are introduced, such as acetylation and two novel acylation modifications—succinylation and crotonylation—to show the effects of acylation on immune cells (Table 2).

### 3.1. Macrophage

Acetylation plays a regulatory role not only in metabolism but also in immune responses. In cases of chronic inflammation and viral infections, protein acetylation modulates the activity and behavior of immune cells, shaping their responses. A notable example is the NLRP3 inflammasome in macrophages, which undergoes acetylation during chronic inflammation. This modification triggers the production of pro-inflammatory cytokines like IL-1β and IL-18 [54]. Additionally, acetylation also affects the ability of the immune system to attack tumors. In tumor-associated macrophages, the acetylation of the transcription factor STAT6 affects their ability to kill tumor cells. STAT6 typically promotes M2 macrophage polarization, which suppresses immune responses [4]. However, the acetyltransferase CBP mediates acetylation at the K383 site, suppressing M2 polarization and thereby enhancing the ability of macrophages to combat tumors [55,56].

Succinylation serves as a link between metabolic shifts and immune responses. Lipopolysaccharide (LPS) exposure significantly raises succinate levels in macrophages, leading to the stabilization of HIF1α and promoting the production of the pro-inflammatory cytokine IL-1β. This highlights the connection between succinate production, succinylation, and the inflammatory response [56]. Additionally, the metabolism shifts in LPS-stimulated macrophages from oxidative phosphorylation to glycolysis, increasing succinate production, and influencing immune responses [57].

As an important epigenetic modification, crotonylation significantly impacts macrophage function, particularly in inflammatory responses and DNA repair. In renal fibrosis, increased histone crotonylation (Kcr) is closely associated with macrophage activation. Specifically, in fibrotic kidneys, the enzyme ACSS2 facilitates crotonylation of histone H3 lysine 9 (H3K9cr) and regulates the expression of the pro-inflammatory cytokine IL-1β. ACSS2 suppresses H3K9cr-mediated IL-1β expression, alleviates macrophage activation, and delays renal tubular cell senescence, presenting a potential therapeutic strategy for renal fibrosis [58]. Moreover, proteins containing YEATS domains, such as AF9 and YEATS2, specifically recognize histone crotonylation modifications, exhibiting approximately seven times greater binding affinity for crotonylation than for acetylation. This specific recognition activates downstream signaling pathways, influencing gene expression and immune responses in macrophages. For example, the YEATS domain of AF9 recognizes crotonylation of lysines 9, 18, and 27 on histone H3 (H3K9cr, H3K18cr, and H3K27cr), thereby promoting gene transcription [52,59].

### 3.2. Acquired Immune Cells

#### 3.2.1. T Cells

During T-cell activation and differentiation, the levels of histone acetylation are markedly altered. An increase in histone acetylation is observed during T-cell activation, particularly in terminally differentiated effector memory T cells (TEMRA). It is closely associated with the stem cell-like properties and terminal differentiation state of T cells. Studies have demonstrated that inhibiting acetylation using the histone acetylation inhibitor C646 modulates the production of effector molecules in T cells, influencing the maintenance of their stem cell-like characteristics and presenting a potential intervention strategy for regulating T-cell functions [68]. Furthermore, the regulation of metabolic pathways is also intimately linked with acetylation modifications in T cells. The activity of mitochondrial isocitrate dehydrogenase is regulated by acetylation, which in turn affects the metabolic status and function of T cells [69]. In CAR-T cells, enhancing mitochondrial metabolism and histone acetylation has been shown to improve antitumor efficacy [70]. Furthermore, acetate salts modulate the acetylation of glyceraldehyde-3-phosphate dehydrogenase (GAPDH) and the differentiation of CD4+ T helper 1 (Th1) cells by altering the levels of acetyl-CoA. This regulation enhances GAPDH activity, aerobic glycolysis, and Th1 polarization, highlighting the role of acetylation in shaping T-cell metabolism and function [60].

Succinylation is critical to the immune environment of tumors, particularly in shaping the phenotype of immune cells like regulatory T cells (Tregs). A comprehensive analysis of succinylation-related enzymes, including CPT1A, KAT2A, SIRT5, and SIRT7, has shown that altering the expression of these enzymes can impact Treg infiltration into tumors. High levels of SIRT7 and low levels of SIRT5 or CPT1A were found to correlate with increased infiltration of FOXP3+ Tregs, contributing to an immunosuppressive tumor environment [61].

#### 3.2.2. B Cells

Moreover, acetylation is a key epigenetic mechanism that regulates B-cell development and function by modulating the expression of various genes and transcription factors. The histone acetyltransferase p300/CBP-associated factor (PCAF) regulates gene expression by catalyzing the acetylation of histone lysine residues and altering the chromatin structure. In immature B cells [62,63], PCAF activity is essential for the control of immunoglobulin synthesis and secretion, a function that has a significant impact on the adaptive immune response [64]. In addition to PCAF, Pax5 is also a key lineage-specific transcription factor in B-cell development [71]. Pax5 regulates genome-wide architecture [72], restricts pre-B-cell proliferation, represses Myc expression, limits cellular metabolism, and promotes cyclic extrusion of immunoglobulin heavy chain (Igh) sites [73,74]. Notably, Pax5 dynamically binds to different genomic regions and regulates the expression of different target genes in both developing and mature B cells [75]. In particular, the NAD+-dependent deacetylase SIRT7 [76] orchestrates B-cell development by specifically deacetylating the K198 site of Pax5. This modification is essential for maintaining the stability and transcriptional activity of the Pax5 protein, revealing a regulatory role for SIRT7 in B-cell development [65].

LYN tyrosine kinase is a non-receptor tyrosine kinase, a member of the Src kinase family, playing a critical role in the regulation of immune cell function [66]. The TCA recycling intermediate fumarate regulates B-cell activation, proliferation, and function through Lyn, a substrate of fumarate that can be succinylated at C381. Since LYN is required for initiating BCR signaling, and its sustained activation further promotes BCR signaling and calcium flux in response to BCR cross-linking, fumarate-induced succinylation of LYN may inhibit B-cell activation and function [67].

Although numerous studies have highlighted the importance and therapeutic potential of acyl modifications in various diseases and even cancer, most of them have focused on classical acyl modifications. Little research has been conducted on novel acyl modifications such as crotonylation and succinylation for the treatment of immune diseases. The diverse functions of specific acyl modifications, along with the complexity of their regulation through enzymatic or non-enzymatic mechanisms, lead to limitations and challenges in preclinical and clinical trials of multiple inhibitors of upstream and downstream pathways of novel acyl modifications.

### 3.3. Clinical Studies on Acylation

In the field of acylation modification, histone deacetylase (HDAC) inhibitors have become the first approved epigenetic drugs. Vorinostat (Zolinza^®^) was approved by the FDA for refractory cutaneous T-cell lymphomas by the mechanism of restoration of histone acetylation levels and modulation of gene expression for antitumor effects [77]. A phase I/II study evaluated the more selective HDAC6 inhibitor ricolinostat (ACY-1215) in combination therapy with bortezomib and dexamethasone. Of the 57 patients treated, the overall response rate (partial response or better) was 29%, and the clinical benefit rate (mild response or better) was 39%. Notably, response rates ranged from 14% to 20% in bortezomib-resistant patients, suggesting that this combination therapy may be a new treatment option for relapsed or refractory patients. The therapy has a favorable safety profile and has shown synergistic antitumor effects, supporting its potential for further clinical development [78].

Romidepsin (Istodax^®^), also an FDA-approved HDAC inhibitor for the treatment of cutaneous and peripheral T-cell lymphomas, has been used to dynamically monitor histone H3 acetylation levels in patient peripheral blood mononuclear cells (PBMCs) in several Phase II and early-phase clinical trials. In a Phase II study involving a dose of 14 mg/m^2^ and intravenous infusions on days 1, 8, and 15, the median increase in histone H3 acetylation in patient PBMCs reached a median 3.0-fold increase at 4 h of dosing and remained elevated 1.85-fold and 1.46-fold at 24 h and 48 h, respectively, suggesting that the drug can sustainably target HDAC activity in vivo [79]. Clinical data also showed that sustained histone acetylation levels were negatively correlated with drug clearance and positively correlated with patient therapeutic response, supporting the use of histone acetylation assays in PBMCs as a pharmacodynamic (PD) biomarker of HDAC inhibitor efficacy [80].

## 4. Conclusions

**Figure 4:**
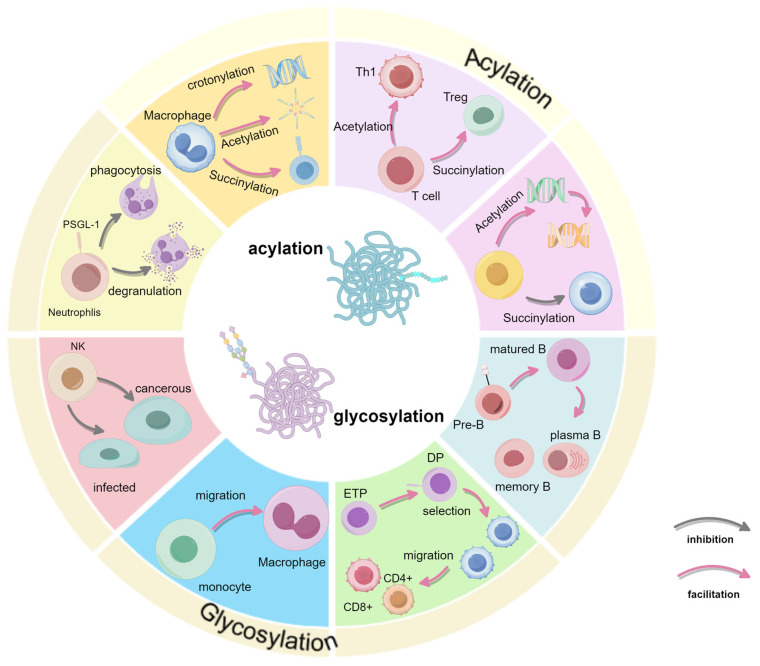
Summary of the full text. Summarizes the effects of glycosylation and acylation on immune cells in terms of development or function.

Immune cell fate decisions are critically influenced by PTMs, which regulate processes such as cell differentiation, activation, and apoptosis. This review has explored the roles of key PTMs—including glycosylation, acetylation, succinylation, and crotonylation—in modulating immune cell functionality. These PTMs enable immune cells to respond effectively to environmental signals, thereby supporting immune homeostasis and precise immune responses (Figure 4).

Therapeutic strategies targeting PTMs are emerging as potential immunomodulatory interventions. For example, manipulating glycosylation has shown promise in enhancing T-cell and B-cell activity, as changes in glycosylation patterns can affect receptor binding and immune cell interactions with antigens. Acetylation and crotonylation, by modifying the histone structure, also play a key role in regulating gene expression in immune cells, providing opportunities to modulate inflammatory responses and potentially improve immunotherapy outcomes [81].

Research on PTMs has become increasingly in-depth in recent years, but there are still some issues that should be addressed in the future. First, the fate of immune cells does not depend on one modification alone, but the mechanism of these interactions has not been clarified. Current research focuses only on single signaling pathways, and the interaction effects of glycosylation and acylation remain largely unexplored in many diseases, including cancer. Second, large-scale studies on PTMs have long been hampered by the lack of suitable analytical techniques. Mass spectrometry is currently the predominant analytical technique; however, the detection of low-abundance modifications (e.g., succinylation) or dynamic transient modifications (e.g., nitrosylation) remains limited. Complete protein structure identification requires more material and analysis time than “simple” identification based on a small number of peptides [82]. Third, the development of targeted drugs against PTMs is still in the initial exploration stage, and clinical applications are still lacking. Despite the remarkable success of drug development that targets protein kinases, histone deacetylases, and ubiquitin ligases, the extreme complexity and dynamic nature of PTMs present major challenges, such as the possibility of off-target effects. Targeting PTM crosstalk will become a powerful strategy in drug research, providing additional drug combinations for the treatment of diseases [83].

In summary, understanding the mechanisms by which PTMs regulate immune cell fate opens promising avenues for novel immune-based therapies. By further investigating these modifications, future research can advance immune modulation strategies, aiming to harness the full potential of PTMs in treating cancer and immune-related disorders.

## Figures and Tables

**Table 1:** Overview of the effects of glycosylation on immune cells.

Immune Cells	Factor	Impact	Effect (+: Positive; −: Negative)	References
Neutrophils	PSGL-1	Cell activation, migration	+	[11]
	AzurocidinCG	Immune response	+	[12,13]
	GlycoRNA	Modulation of the inflammatory response	+ +	[14,15]
NK cells	Siglec-7, Siglec-9	Killing activity	−	[16,17]
	CD16a	Killing activity	+	[18]
Macrophages	Agglutinin (DC-SIGN)	Immune response	−	[10]
	Pro-inflammatory cytokine	Immune response	+	[13,19]
	CD24, CD47	Phagocytosis	−	[20]
T cells	OGT	Cell development	−	[21]
	Mgat1	Cell development	−	[22,23]
	IL-2	Cell differentiation Treg	+	[24]
	PD-1	Cell activation	−	[25,26]
B cells	μ-heavy chain	Pre-BCR	+	[27]
	Galectin-1	Cell maturation	+	[28]
	CD19	Cell development	+	[29]

**Table 2:** Overview of the effects of acylation on immune cells.

Immune Cells	Acylation Type	Factor	Impact	Effect	References
Macrophages	Acetylation Succinylation Crotonylation	NLRP3	Inflammation	+	[54]
		STAT6	Cell polarization	+	[4]
		CBP	Cell polarization	−	[55,56]
		LPS	Inflammatory	+	[57]
		H3K9cr/ACSS2	Cell activation	+	[58]
		AF9/YEATS2	Gene transcription	+	[59]
T cells	Acetylation Succinylation	Acetate	Cell metabolism	+	[60]
		Enzyme (KAT2A, SIRT7)	Cancer progress	+/−	[61]
B cells	Acetylation	PCAF	Cell function	+	[62,63,64]
		Pax5/SIRT7	Cell development	+	[65]
	Succinylation	LYN	BCR signal transduction	+	[66,67]
